# Transcription-Coupled Repair Promotes the Retention of Mutations in Coding Regions During Replication Stress

**DOI:** 10.3390/ijms27031154

**Published:** 2026-01-23

**Authors:** Evelyn Zambrano, Cristopher Fierro, Fernanda Morales, Marcia Manterola, Arnaldo Marin, Ricardo Armisen, Katherine Marcelain

**Affiliations:** 1 Departamento de Oncología Básico Clínico, Facultad de Medicina, Universidad de Chile, Santiago 8380453, Chile; evelyn.zambrano@ug.uchile.cl (E.Z.); fierrocristopher@gmail.com (C.F.); fernmorales@ug.uchile.cl (F.M.); arnaldo.marin@uchile.cl (A.M.); 2 Centro para la Prevención y el Control del Cáncer (CECAN), Facultad de Medicina, Universidad de Chile, Santiago 8380453, Chile; 3 Núcleo Interdisciplinario de Biología y Genética, ICBM, Facultad de Medicina, Universidad de Chile, Santiago 8380453, Chile; mmanterola@uchile.cl; 4 Centro de Estudios Traslacionales en Estrés y Salud Mental (C-ESTRES), Universidad de Valparaíso, Valparaíso 2340000, Chile; 5 Centro Genética y Genómica, Instituto de Ciencias e Innovación en Medicina, Clínica Alemana Universidad del Desarrollo, Santiago 7610658, Chile; rarmisen@udd.cl

**Keywords:** ERCC6 (CSB), transcription-coupled nucleotide excision repair (TC-NER), replication stress, genomic instability, mutagenesis

## Abstract

Replication stress (RS) is a primary driver of genomic instability in cancer, yet the contribution of transcription-coupled repair (TC-NER) to this process remains unclear. Here, we investigate how the TC-NER factor ERCC6 (CSB) shapes mutational landscapes under RS. We found that ERCC6 deficiency biases early damage signaling toward a 53BP1-mediated response, ultimately leading to senescence. Conversely, ERCC6-proficient cells prioritize survival and proliferative recovery but at the expense of distinct genomic alterations. Whole-exome sequencing reveals that ERCC6 proficiency is associated with the retention of stress-induced mutations specifically within coding regions of transcriptionally active loci, whereas ERCC6-deficient cells accumulate variants primarily in intergenic regions. These findings suggest that while ERCC6 safeguards transcriptional continuity during RS, its activity is associated with a biased retention of stress-induced mutations within coding regions in the surviving cell population. These findings reveal a previously unrecognized link between transcription-coupled repair and mutation distribution in human cells, linking TC-NER to context-dependent somatic evolution and tumor heterogeneity.

## 1. Introduction

Replication stress (RS) is a critical source of genomic instability in cancer, driving the accumulation of mutations and chromosomal rearrangements that promote tumor progression [1]. RS arises when replication forks encounter obstacles caused by DNA lesions, insufficient replication factors, oncogene activation, or conflicts with the transcription machinery [2,3,4,5,6,7]. Transcription–replication conflicts (TRCs) can generate R-loops [8]. These are three-stranded structures formed by an RNA–DNA hybrid and a displaced single-stranded DNA strand. These structures can stall replication forks and become sources of DNA damage if not properly resolved [9,10].

R-loops can be processed through the transcription-coupled nucleotide excision repair (TC-NER) pathway. TC-NER factors, including ERCC6, operate in cellular environments where R-loop-associated damage frequently coexists with strand breaks, linking transcription-coupled repair with genome instability under RS [11,12,13].

In addition to canonical functions associated with TC-NER, ERCC6 is involved in other DNA damage repair mechanisms. At DNA double-strand breaks (DSBs), ERCC6 has been shown to influence pathway choice by favoring homologous recombination (HR) through ATM- and CDK2-dependent displacement of RIF1 and 53BP1 [13,14]. ERCC6 also contributes to the resolution of transcription-blocking DNA–protein crosslinks [15,16,17], facilitates repair of oxidative DNA lesions within transcribed chromatin [18,19,20,21,22], and cooperates with PARP1 and PARP2 in single-strand break repair [23,24,25]. Notably, the repair functions of ERCC6 are not uniformly protective across all cellular contexts.

More recent investigations have conclusively established a vital role for ERCC6 in the direct management of replication stress, separate and independent from global TC-NER. ERCC6 is detected at ongoing replication forks in unperturbed cells but becomes significantly enriched at stalled forks upon replication stress [26]. Under mild replication stress conditions, ERCC6 binds RPA at stalled forks and acts to restrain fork progression [27]. Furthermore, ERCC6 has been implicated in fork reversal and has been shown to regulate fork restart pathway choice following high-dosage, CPT-induced fork stalling; specifically, it promotes RAD52-dependent break-induced replication (BIR) while inhibiting Alt-EJ, NHEJ, and fork repriming [26,28]. This preference for BIR is particularly consequential because BIR DNA synthesis is intrinsically inaccurate over the entire path of the replication fork, with frameshift mutagenesis rates up to 2800-fold higher than during normal replication [29].

In bacteria, the ERCC6 functional homolog Mfd promotes stress-induced mutagenesis, both under non-growing or antibiotic stress conditions and at replication–transcription conflicts via an error-prone NER pathway and R-loop formation [30,31,32,33,34,35]. However, whether ERCC6 exerts a comparable pro-mutagenic role in human cells under RS remains unclear.

Evidence from mammalian systems indicates that TC-NER is not simply a faster repair pathway, but plays a non-redundant role in the removal of specific types of transcription-blocking DNA lesions, including certain oxidatively induced lesions that GG-NER handles poorly [36,37,38]. Consequently, damage processing is unlikely to be uniformly distributed across the genome but instead concentrated within transcriptionally active loci.

The genomic distribution of somatic mutations is functionally relevant, as alterations affecting coding regions in oncogenes and tumor suppressor genes give rise to driver events that confer selective growth advantages, ultimately shaping clonal architecture and tumor evolution [39,40]. Moreover, several anticancer therapies induce RS as part of their mechanism of action [41,42,43]. In line with this, CRISPR-based screening in patient-derived organoids has identified ERCC6 as a determinant of cisplatin resistance in osteosarcoma [44]. Understanding how ERCC6 affects both survival and mutation patterns under RS, therefore, has direct implications for therapeutic response.

Given that under replication stress, stalled forks are likely to increase transcription–replication conflicts (TRCs), particularly in transcriptionally active genes, we hypothesized that ERCC6-mediated resolution might influence mutational outcomes in these regions. In this work, we studied the involvement of ERCC6 in the cellular response of human fibroblasts (ERCC6 wild type and ERCC6-null) exposed to 2 mM HU for 1 h, 4 h, and 24 h to induce acute, mild, and severe replication stress, respectively. We comprehensively explored cellular fate and mutational patterns related to TC-NER-associated ERCC6 function. To this end, we evaluated DNA damage signaling, transcriptional remodeling, and mutational burden and distribution through immunofluorescence imaging, RNA sequencing, and whole-exome sequencing, respectively. Our findings suggest that while ERCC6 supports TRC resolution and replication fork restart, its activity is associated with a biased retention of stress-induced mutations within coding regions in the surviving cell population. These findings reveal a previously unrecognized link between transcription-coupled repair and mutation distribution in human cells.

## 2. Results

### 2.1. Hydroxyurea-Induced Replication Stress in a TC-NER-Deficient Model

ERCC6_K337X cells showed functional loss of TC-NER, as demonstrated by their hypersensitivity to Illudin S, a DNA-damaging agent specifically repaired through this pathway (Figure S1). This confirms that the ERCC6_K337X line is a valid model for studying TC-NER deficiency.

**Figure 1:**
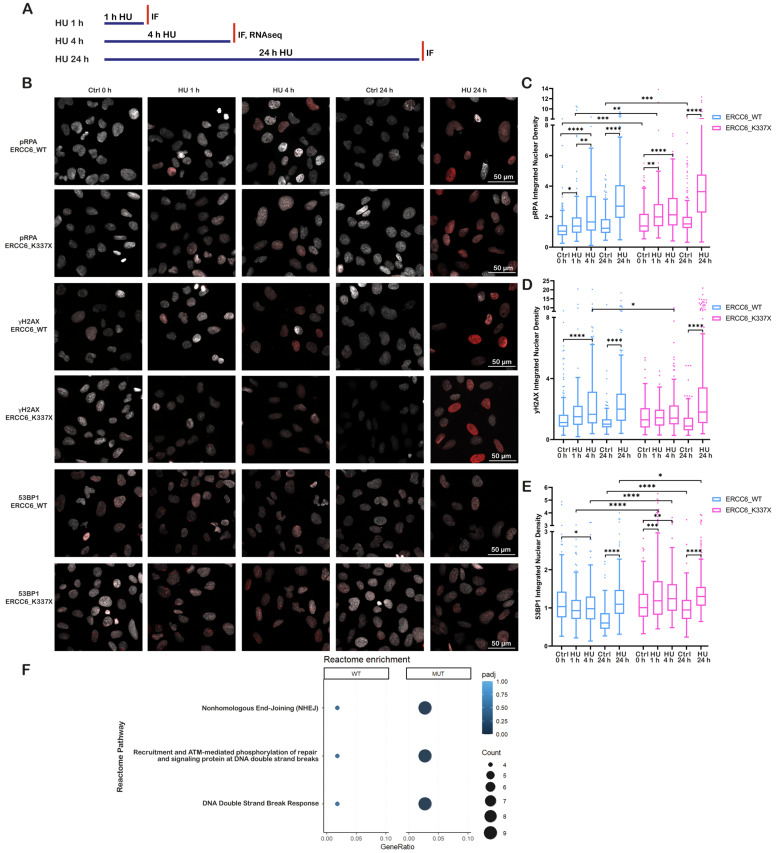
ERCC6 deficiency alters DNA damage signaling dynamics under replication stress. (**A**) Schematic overview of HU exposure conditions and experimental readouts used in this figure. Cells were exposed to HU for 1 hour (h), 4 h, or 24 h, and independent analyses were performed as indicated. (**B**) Representative immunofluorescence (IF) images of ERCC6_WT and ERCC6_K337X fibroblasts under control conditions (0 h (hours), 24 h), and HU exposure (1 h, 4 h, 24 h, 2 mM). Nuclei are shown in gray and pRPA, γH2AX and 53BP1 in red. Scale bar, 50 μm. (**C**–**E**) Integrated nuclear signal density (IntDen) for pRPA, γH2AX and 53BP1. Box-and-whisker plots follow the Tukey method. Data represent mean ± SD from three independent experiments (n > 180 nuclei). (**F**) Reactome pathway enrichment analysis of differentially expressed genes in ERCC6_WT and ERCC6_K337X cells under HU. Selected pathways relevant to DSBs response. Statistics: (**C**–**E**) Welch’s ANOVA with Brown–Forsythe correction, followed by Games–Howell post hoc test. Significance levels: *p* < 0.05 (*), *p* < 0.01 (**), *p* < 0.001 (***), *p* < 0.0001 (****). (**F**) Reactome pathway analysis from RNA-seq experiments. Significance threshold: *p* adj < 0.05. Pseudoreplicates (n = 1 per condition) were used for pathway-level comparisons, analysis (*p* adj, BH (Benjamini–Hochberg) correction).

Basal levels of replication protein A (RPA) were comparable between ERCC6_WT and ERCC6_K337X cells (Figure S2), indicating similar steady-state fork dynamics. As outlined in the experimental scheme (Figure 1A), phosphorylation of RPA at serine 32 (pRPA) increased upon treatment with 2 mM HU for 1, 4, and 24 h in both genotypes (Figure 1B,C), confirming that HU induces RS and fork stalling to a similar extent in WT and ERCC6-deficient cells.

### 2.2. ERCC6 Deficiency Compromises Transcription–Replication Conflict (TRC) Resolution and Promotes Early NHEJ Activation Under Replication Stress

ERCC6_WT cells showed an increase in γH2AX levels, following short (1 and 4 h) and long (24 h) exposure to HU, with the strongest signal observed after 24 h of treatment (Figure 1B,D), consistent with a fork collapse, DSBs generation, and active repair. In contrast, ERCC6_K337X cells did not display a significant increase in γH2AX signal after short-term HU exposure, with a significant increase observed only after 24 h.

To evaluate if these differences observed after short-term exposure were due to differences in the resolution of the TRCs, RNAPII–PCNA interactions were detected by proximity ligation assay (PLA), as a proxy for identifying TRCs upon HU treatment (Figure S3). No significant differences in the number of PLA foci per nucleus were observed between control and HU-treated WT and mutant cells under any condition. Nevertheless, ERCC6_K337X cells exposed to 2 mM HU for 4 h exhibited a significant increase in the median volume of nuclear PLA foci relative to both control and WT cells, consistent with a reduced TRCs resolution capacity in ERCC6-deficient cells.

Thus, we evaluate if the lack of increase in γH2AX foci after short-term HU exposure was related to DSB repair. After 24 h of HU exposure, both ERCC6_WT and K337X cells showed elevated 53BP1 levels. However, short HU exposure (1–4 h) resulted in a marked 53BP1 recruitment in ERCC6_K337X cells, whereas ERCC6_WT cells showed a slight reduction compared to control (Figure 1B,E). Reactome pathway enrichment analysis of genes differentially expressed (DEGs) in HU-treated WT and mutant cells further supported these findings, showing an over-representation of pathways associated with NHEJ only in ERCC6_K337X cells after 4 h of HU treatment (Figure 1F).

Together, these results suggest that ERCC6 deficiency limits the resolution of TRCs and shifts the damage response toward early NHEJ-mediated repair with short-time RS.

### 2.3. ERCC6 Deficiency Impairs Replication Dynamics and Promotes Senescence Under Replication Stress

**Figure 2:**
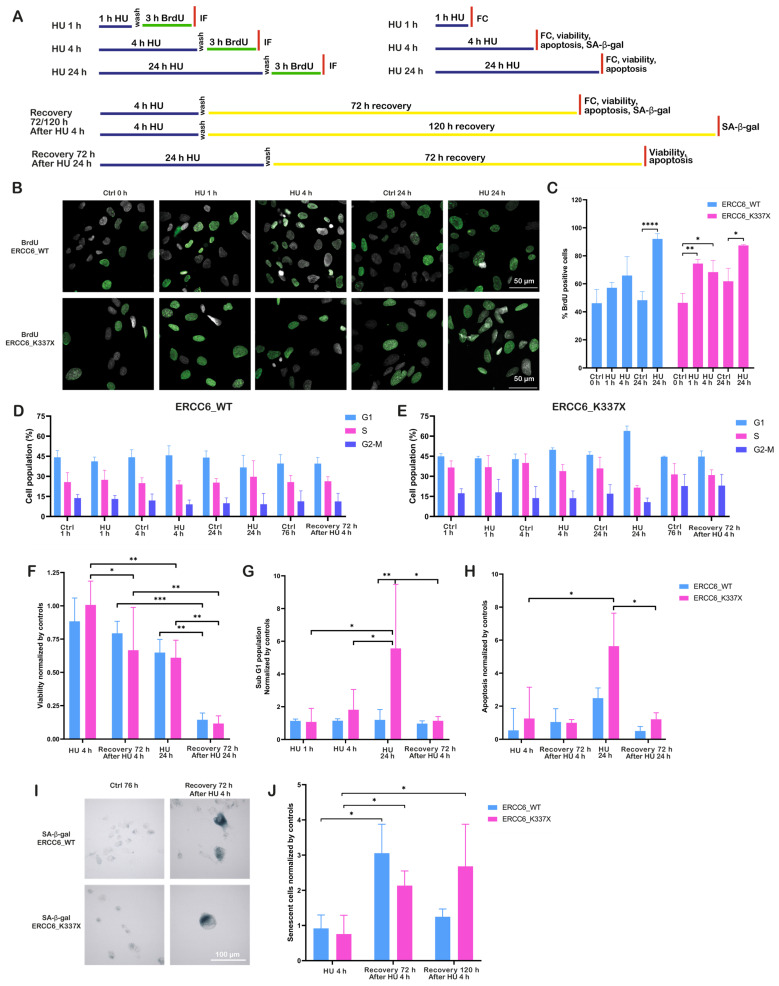
ERCC6 determines replication restart and senescence outcomes. (**A**) Schematic overview of HU exposure conditions, recovery periods, and experimental readouts used in this figure. Cells were exposed to HU for 1 h, 4 h, or 24 h, followed by washout and recovery where indicated; independent analyses were performed at the specified time points. FC (Flow Cytometry). (**B**) Representative BrdU IF images of ERCC6_WT and ERCC6_K337X cells under control conditions and after HU exposure (1 h, 4 h, 24 h). Nuclei in gray, BrdU in green. Scale bar, 50 μm. (**C**) Percentage of BrdU-positive nuclei. Data represent mean ± SD from three independent experiments, 4–5 fields each (n = 12–15 images). (**D**,**E**) Cell-cycle profiles (G1, S, G2/M) assessed by propidium iodide staining in ERCC6_WT (n = 4). (**D**) and ERCC6_K337X (n = 3). (**E**) Cells under control, HU (1 h, 4 h, 24 h), and 72 h post-HU recovery. No statistical testing was applied. (**F**) Cell viability expressed as fold-change relative to the respective untreated control, measured after HU (4 h and 24 h) and after 72 h recovery (n = 3). (**G**) Sub G1 population expressed as fold-change relative to the respective untreated control, measured after HU (4 h and 24 h) and after 72 h recovery (n = 3). (**H**) Apoptosis levels expressed as fold-change relative to control, under the same HU exposure and recovery conditions (n = 3, except 72 h recovery after HU 24 h (n = 2)). (**I**) Representative images of SA-β-Gal staining. Senescent cells appear in blue. Scale bar, 100 μm. (**J**) Quantification of senescent cells normalized to control (fold-change) at HU 4 h, and at 72 h and 120 h recovery (n = 3). Statistics: Two-way ANOVA with Tukey’s multiple comparisons test (**C**,**G**,**H**,**J**) and two-way ANOVA with Benjamini, Krieger y Yekutieli (BKY) false discovery rate (FDR) correction for multiple comparisons (**F**). Plots use Tukey distribution with all the replicates. Significance levels: *p* < 0.05 (*), *p* < 0.01 (**), *p* < 0.001 (***), *p* < 0.0001 (****).

As outlined in the experimental scheme (Figure 2A), DNA synthesis following RS was assessed in cells exposed to a 3 h BrdU pulse immediately after HU washout (RS release). BrdU incorporation assays revealed distinct DNA synthesis dynamics between ERCC6_WT and ERCC6_K337X cells after release from HU-induced RS. In ERCC6_WT cells, the proportion of BrdU-positive nuclei increased gradually after prolonged exposure to HU, being significantly higher than control only in cells released after 24 h HU treatment (Figure 2B,C). In contrast, ERCC6_K337X cells exhibited a significant increase in BrdU incorporation in cells released after short (1 h and 4 h) and long (24 h) HU treatment, consistent with a G1/S accumulation and rapid resumption of DNA synthesis in the absence of ERCC6. Cell-cycle profiling supported this interpretation (Figure 2D,E). HU did not induce major changes in the cell-cycle distribution of ERCC6_WT cells after short HU treatment. An increase in S-phase cells can be evidenced only after 24 h of treatment. On the other hand, ERCC6_K337X cells showed a significant enrichment of G1-cells after 4 and 24 h of treatment, consistent with replication fork collapse followed by cell-cycle arrest.

This difference in replication recovery was reflected in cell fate outcomes. Prolonged HU treatment (24 h) induced an approximately 30% decrease in viability in both genotypes relative to untreated controls (Figure 2F), which further declined to less than 20% after 72 h of recovery. On the other hand, a significant decrease in viability after 72 h recovery from short-term HU exposure (4 h), was observed only in ERCC6-deficient cells (Figure 2F). Consistent with this pattern, increased apoptotic cell death was observed only after prolonged HU exposure and primarily in ERCC6-deficient cells (Figure 2G,H), whereas short-term HU treatment did not result in a significant apoptotic response. The decrease in viability in cells that survive the 4 h HU exposure is accompanied by an increase in senescent cells (Figure 2I,J). ERCC6_WT cells displayed a transient increase in SA-β-Gal activity at 72 h post-recovery, which declined by 120 h, consistent with temporary growth arrest followed by proliferative recovery. In contrast, ERCC6_K337X cells exhibited a progressive and persistent increase in senescent cells, indicating stable growth arrest as a terminal outcome of unresolved RS. Together, these findings indicate that ERCC6 supports efficient replication restart and limits long-term growth arrest under RS. In its absence, cells experience delayed replication completion, replication fork collapse, and would fail to recover proliferative capacity, resulting in apoptosis or persistent senescence.

### 2.4. Divergent Transcriptional Programs Emerge Under Replication Stress Depending on ERCC6 Status

**Figure 3:**
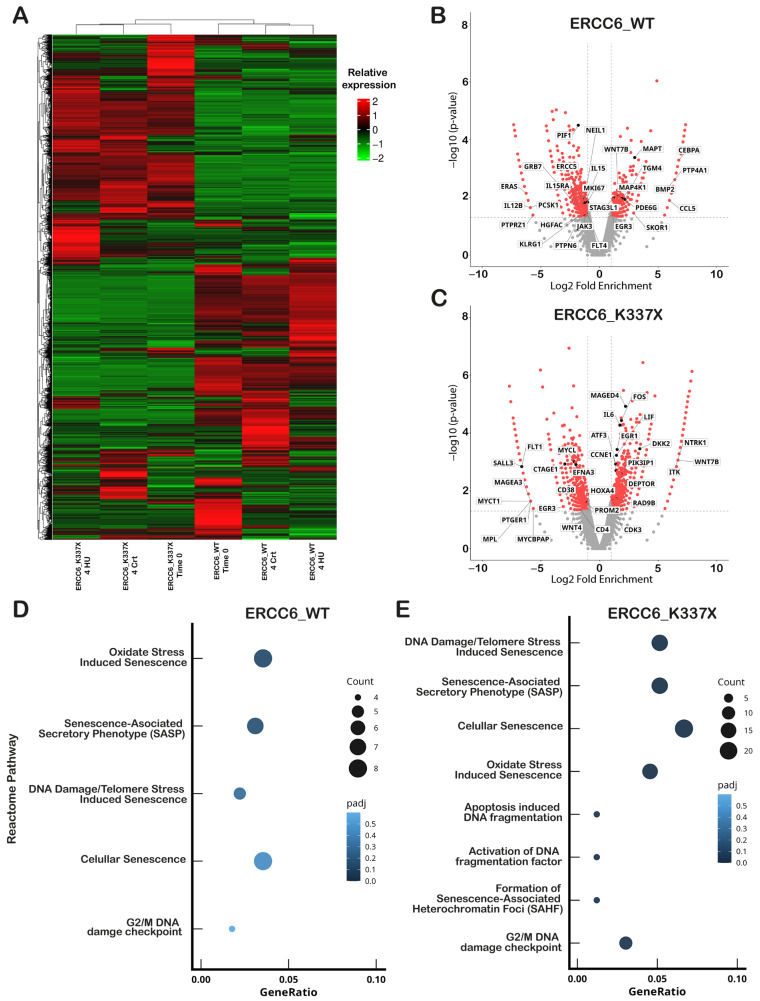
ERCC6 status shapes transcriptional responses under replication stress. (**A**) Hierarchical clustering heatmap of differentially expressed genes (DEGs) in ERCC6_WT and ERCC6_K337X cells across three conditions: basal (T0), HU (4 h) and matched controls (4 h Ctrl). Each row represents a gene and each column a sample. Color scale represents expression normalized by log2(FPKM + 1) and homogenized row by Z-score (red = high, green = low), hierarchical clustering was performed on the log2(FPKM + 1) values to group genes with similar expression patterns. (**B**) Volcano plot showing DEGs in ERCC6_WT cells under HU versus control. (**C**) Volcano plot showing DEGs in ERCC6_K337X cells under HU versus control. In (**B**,**C**), log_2_ fold change is plotted against −log_10_(*p* value); significantly regulated genes (*p* value < 0.05) are highlighted with red. Genes annotated to cancer pathways are indicated. Differential expression was determined using edgeR with pseudoreplicates (n = 1 per condition). (**D**,**E**) Reactome pathway enrichment analysis of differentially expressed genes in ERCC6_WT (**D**) and ERCC6_K337X (**E**) cells under HU. Selected pathways relevant to RS response, DNA repair signaling, cell-cycle control and senescence are shown. Reactome significance threshold: *p* adj < 0.05 (BH correction). Pseudoreplicates (n = 1 per condition) were used for pathway-level comparisons.

To further understand the differences in cell fate in ERCC6 null cells exposed for 4 h to HU, we analyzed the HU-induced differential transcriptional program in WT and deficient cells. Hierarchical clustering revealed a clear transcriptional separation between ERCC6_WT and ERCC6_K337X cells under basal and HU-induced RS conditions (Figure 3A). The two genotypes showed substantial differences in the total number of expressed genes; ERCC6_K337X cells displayed a broader transcriptional response to HU (2039 vs. 1247 DEG), with a predominance of upregulated genes (1128 vs. 496, in mutant and WT cells, respectively) (Figure S4). This indicates a distinct transcriptional program activation associated with ERCC6 status. Among DEGs, there are genes previously linked to oncogenic pathways and DNA repair. Some of them are labeled in Figure 3B,C. For instance, ERCC6_WT cells activated adaptive pro-survival genes, including WNT7B↑, MAP4K1↑, BMP2↑, MKI67↓, PTPN6↓, PTPRZ1↓ while suppressing senescence- and immune surveillance-associated cytokines such as IL12B, IL15/IL15RA, and KLRG1. Notably, NEIL1 and ERCC5 (XPG) were downregulated after HU exposure, consistent with an efficient early damage resolution and proliferative recovery. In contrast, ERCC6_K337X cells upregulated stress and inflammatory response genes (IL6↑, LIF↑, FOS↑, EGR1↑, ATF3↑) together with checkpoint and DNA damage regulators (RAD9B↑, DEPTOR↑, PIK3IP1↑), while downregulating proliferation- and metabolism-supporting genes (MYCL↓, PROM2↓, MAGEA3↓, MPL↓).

To determine whether these transcriptional differences reflected RS-specific effects or a pre-existing ERCC6-dependent state, we next examined differential expression under basal (untreated) conditions (ERCC6_WT_Control vs. ERCC6_K337X_Control) (Figure S5a). Genes differentially expressed upon HU exposure were largely distinct from those identified under basal conditions; moreover, for genes shared between basal and HU-treated states (e.g., WNT7B, IL15RA, MYCL, CCNE1), regulation was not necessarily in the same direction. In addition, no basal differential expression was detected in DNA damage and repair genes associated with RS, including RAD9B and NEIL1, suggesting that their transcriptional modulation is specifically associated with the replication stress context rather than with the ERCC6-dependent basal state (Figure S5).

Reactome pathway enrichment analysis of HU-induced DEGs further supported long-term cell fate outcomes observed in WT and ERCC6 null cells, showing an over-representation of senescence and apoptosis-related pathways in ERCC6_K337X cells after 4 h of HU treatment (Figure 3D,E). In contrast, Reactome pathway analysis of significantly DEGs under basal conditions revealed a significant enrichment of only one subset of senescence-associated pathways in ERCC6-deficient cells: DNA Damage/Telomere Stress-Induced Senescence (Figure S5b). These results indicate that ERCC6 deficiency is associated with a senescence-prone transcriptional state already present in the absence of RS. However, broader senescence-related annotations (such as Cellular Senescence, SAHF (Senescence-Associated Heterochromatin Foci) formation, or apoptosis-related DNA fragmentation pathways), as well as canonical DNA damage response pathways (including NHEJ and ATM-mediated signaling), did not reach statistical significance under basal conditions. These findings suggest that ERCC6 deficiency primes specific senescence-associated transcriptional axes, whereas RS exposure further amplifies and expands these pathways and engages additional damage-associated responses.

Collectively, these data support a model in which ERCC6_WT cells that survive a mild HU-induced RS would continue proliferating, whereas ERCC6_K337X cells would undergo a transcriptionally enforced arrest.

### 2.5. ERCC6 Status Determines Distinct Mutational Patterns and Expression–Mutation Coupling Under Replication Stress

The results described above suggest that ERCC6_WT cells can resolve TRCs and stalled forks efficiently. To evaluate whether TC-NER-dependent resolution of the RS is associated with mutagenesis, we performed whole-exome sequencing (WES) in cells exposed to HU for 4 h and in cells exposed for 4 h, followed by 72 h recovery in fresh medium. After 4 h of treatment, mutations are in fact technical sequencing artifacts resulting from HU- and/or TRCs-induced DNA damage. On the other hand, unrepaired and misrepaired DNA damage is expected to be transformed in real mutations after 72 h recovery, although we cannot rule out the presence of persistent DNA damage at this point.

**Figure 4:**
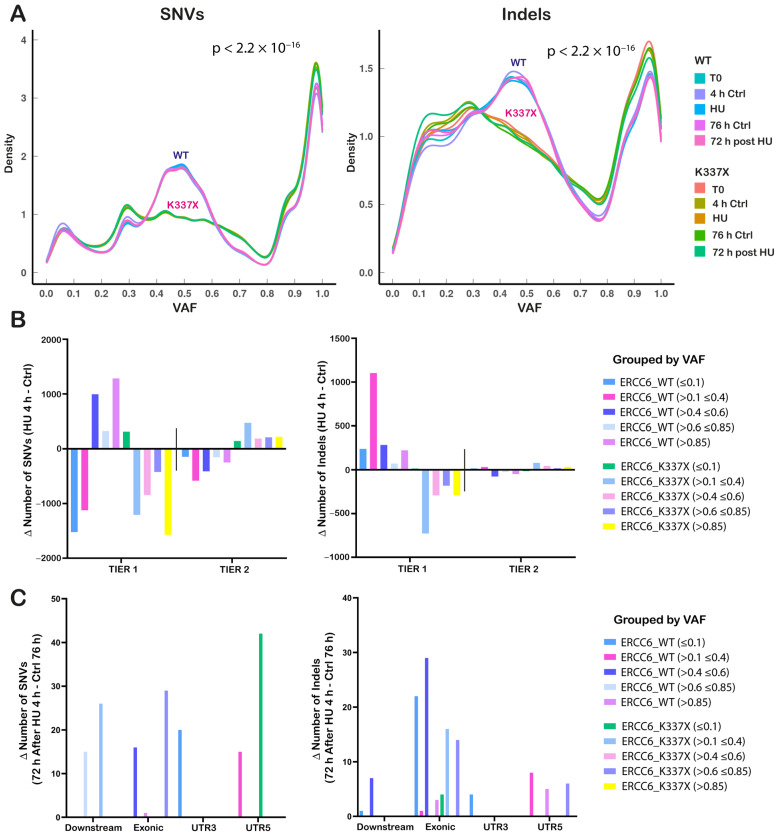
Replication stress differentially redistributes mutation burden by genomic location depending on ERCC6 status. (**A**) Variant Allele Frequency (VAF) distributions of SNVs (Single Nucleotide Variants) (left) and Indels (insertion-deletion) (right) across ERCC6_WT and ERCC6_K337X cells under basal conditions (T0), HU (4 h), matched controls (4 h Ctrl), recovery (72 h post-HU), and its corresponding control (72 h Ctrl). Differences in distribution between WT and K337X cells were assessed using the Kolmogorov–Smirnov test. (**B**) Mutations grouped into Tier 1 (genic regions: exonic, intronic, UTRs, splicing-associated, upstream/downstream) and Tier 2 (intergenic) categories and further stratified by variant allele frequency ranges (<0.1; 0.1–0.4; 0.4–0.6; 0.6–0.85; >0.85). Bars represent the Δ mutation count (HU 4 h–Ctrl 4 h) for WT and K337X. (**C**) SNVs (left) and Indels (right) with Δ mutation counts restricted to specific genomic categories that showed HU-dependent changes (Downstream, Exonic, UTR3, UTR5). Values represent Δ positive of 72 h recovery after 4 h HU–72 h Ctrl.

Overall, the distribution of variants (SNVs and Indels) by allelic frequency differed between ERCC6_WT and mutant cells, independent of the experimental condition (Figure 4A). In WT cells, SNVs were enriched around VAF ≈ 0.5 and in the high-frequency range (~0.8–1.0), consistent with stable clonal representation. In contrast, ERCC6_K337X cells showed a broader VAF distribution in the 0.2–0.8 range, indicating an increased sub-clonal diversification. The same differences in distribution by VAFs between WT and ERCC6_K337X cells were observed for Indels; however, in this case, the distribution of Indel VAFs varied with the experimental condition, particularly in variants with VAFs < 0.3, with the highest frequency in ERCC6_K337X cells after 72 h HU recovery.

The mutational burden induced by HU was calculated as the difference in the total number of variants detected between HU-treated and untreated cells for each genotype and condition. SNVs and Indels were stratified according to their position relative to genes into tier 1 (gene-proximal: coding sequences, UTRs, and annotated ncRNAs) and tier 2 (intergenic) regions (Figure 4B). Following HU exposure, ERCC6_WT cells accumulated DNA damage (known as mutations) predominantly in tier 1 regions, while ERCC6_K337X cells showed an enrichment in intergenic (tier 2) regions. Consistently, variant calling performed on RNA-seq data revealed a higher number of expressed variants in ERCC6_WT cells and a reduced number in ERCC6_K337X cells following HU exposure (Figure S6a,b).

An overall reduction in mutation counts was detected after 72 h of recovery in both ERCC6 WT and deficient cells. Since no massive cell death was observed at this point (Figure 2F–H), this reduction in the number of mutations may be a result of DNA repair. Nevertheless, only mutations classified as tier 1 were detected in the surviving population of cells, with an enrichment of SNVs in the 5′UTR region in ERCC6_K337X cells, and exonic Indels in WT cells (Figure 4C).

**Figure 5:**
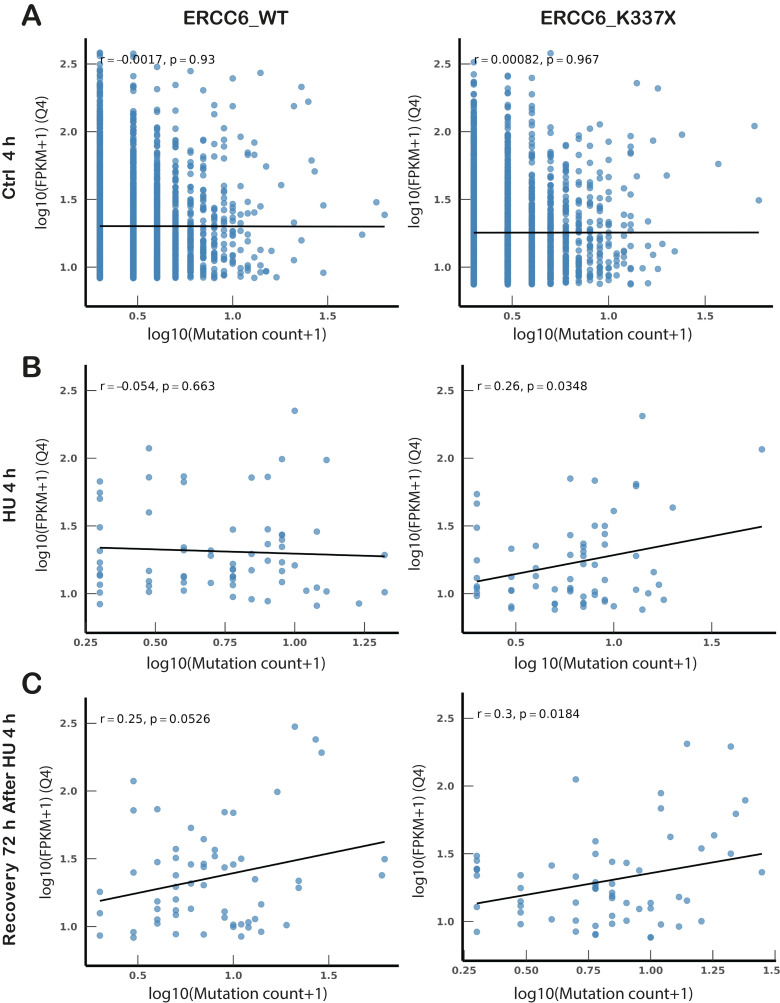
Transcription-dependent mutation patterns differ between ERCC6-proficient and deficient cells. (**A**–**C**) Spearman correlation between gene transcriptional activity (expressed as log10(FPKM + 1)) and mutation counts (expressed as log10(mutation count + 1)) for highly expressed genes (top expression quartile, Q4) in control (**A**), HU (**B**) and recovery (**C**) conditions. In (**B,C**), only genes showing Δ ≥ 3 mutations relative to control were included, isolating stress-induced mutation gains. (**C**) Recovery (72 h post-HU), with mutation changes referenced to matched 72 h Ctrl, while expression corresponds to the HU (4 h) transcriptional state, reflecting the transcriptional environment in which mutagenesis was established. Rho and *p* values indicate Spearman correlation significance. WES and RNA-seq were performed on a single biological sample per condition (n = 1). Statistical comparisons were conducted at the variant level.

The distribution of mutations relative to the genes is consistent with the presence of DNA damage or misrepair following TC-NER-dependent TRCs resolution. To assess whether the presence of mutations was related to the transcriptional activity of the genes at the time of HU-induced RS, these were classified in quartiles according to their expression levels. Correlation between mutation counts and gene expression (FPKM) was assessed in highly expressed genes (Q4) (Figure 5A–C). No correlation was observed under basal (control) conditions, in WT or mutant cells. In WT cells, the HU-induced mutations (4 h HU) are distributed randomly, independent of the transcriptional activity of the gene (Figure 5B), consistent with the resolution of TRCs at this point. After 72 h recovery, most damage was repaired and a weak, non-significant correlation between persistent damage (and/or mutations) and gene expression was observed (r = 0.25, *p* = 0.0526). In contrast, a significant positive correlation was observed between the amount of mutations and gene expression levels in ERCC6_K337X cells after 4h HU and after 72 h of recovery, suggesting that transcriptionally active loci become preferential targets for DNA damage when ERCC6 is absent. These results are consistent with unrepaired DNA damage enriched in unresolved TRCs exacerbated by ERCC6 deficiency.

Together, these findings reveal that ERCC6 status determines both the genomic distribution and the transcriptional dependency of mutagenesis under RS.

## 3. Discussion

Our results suggest that ERCC6 shapes the early DNA damage response to HU-induced RS. Notably, the reduced or absent γH2AX signal after short HU exposure in ERCC6-deficient cells is best explained by a faster 53BP1-driven repair response that promotes c-NHEJ, leading to rapid damage processing and accelerated γH2AX dephosphorylation [13,45,46]. This is consistent with biochemical and genetic evidence showing that 53BP1/RIF1 biases DSB pathway choice toward NHEJ [45], and that CSB/ERCC6 loss shifts repair away from HR toward NHEJ [13]. Moreover, γH2AX clearance during the fast-kinetic phase of DSB repair is driven by PP4-mediated dephosphorylation [46,47]. A fast-kinetic phase could plausibly explain why low γH2AX in ERCC6_K337X cells does not necessarily indicate the absence of damage, but rather reflects altered repair dynamics, with early NHEJ engagement likely compensating for the inability to resolve transcription-associated lesions through TC-NER. Although our data strongly support this model, they do not establish a single mechanistic route, and additional work will be required to dissect specific intermediates generated during TC-NER-associated DSB processing under RS.

Additionally, an increased RNAPII–PCNA proximity signal in ERCC6-deficient cells under HU suggests impaired TRC clearance, consistent with the role of TC-NER proteins in resolving R-loops and polymerase collisions at stalled forks [12,48]. Persistent TRCs are known to generate transcription-blocking lesions that require TC-NER-dependent processing; therefore, differences in TRC resolution are expected to contribute to differences in where RS-associated mutations accumulate and in how cells recover from damage. These results support that TRCs represent a significant source of unresolved lesions in ERCC6-deficient cells; the transcription-dependent mutation patterns observed are compatible with this interpretation.

Notably, ERCC6_WT cells accumulated more mutations in coding regions following short HU exposure and exhibited a transient increase in senescence at 72 h, which declined by 120 h, indicating reversible growth arrest and recovery of proliferative capacity. This observation is consistent with the idea that transcription-coupled repair under stress conditions may be associated with the retention of mutations under those conditions, consistent with models described in bacterial systems [49]. By contrast, ERCC6_K337X cells exhibited reduced viability, not attributable to apoptosis, together with a progressive accumulation of senescent cells over time, consistent with stable proliferative arrest, a pattern also reported under oxidative stress conditions [50]. Importantly, hereditary premature aging disorders characterized by high RS, such as Cockayne syndrome (CS), do not exhibit an increased cancer incidence, unlike XPC-deficient NER disorders, which show strong tumor predisposition [51,52]. Our data provide a mechanistic framework for this distinction. When ERCC6/TC-NER is functional, cells attempt to maintain transcriptional continuity under RS, but at the cost of being associated with the retention of RS-associated mutations. In contrast, when ERCC6 is absent, cells fail to tolerate RS and preferentially enter senescence, limiting the proliferative capacity of damaged, mutation-bearing cells.

These findings imply that TC-NER is essential for survival and transcriptional continuity but may increase the risk of protein-affecting mutations in dividing cells under RS. Understanding this duality is particularly relevant in cancer biology [53], where RS and transcriptional hyperactivation coexist [54,55]. Although this study was performed in non-transformed human cells, investigating how mutagenesis emerges under RS in initially normal cells is essential for understanding the earliest molecular events that initiate genomic instability and tumorigenesis. Tumors do not arise in pre-existing cancer cells but evolve from genomically stable progenitors that progressively accumulate DNA damage and mutations. In this context, ERCC6 emerges as a key double-edged modulator of genomic maintenance under RS.

## 4. Materials and Methods

### 4.1. Cell Lines and Induction of Replication Stress (RS)

Human fibroblasts GM00637 and GM16095 were obtained from the Coriell Cell Repository (Coriell Institute for Medical Research, Camden, NJ, USA)). GM16095 harbors a truncating mutation (ERCC6_K337X) resulting in functional loss of ERCC6. GM00637 is the wild type for ERCC6 (ERCC6_WT). Cells were cultured according to the manufacturer’s instructions in DMEM/F12 (Hyclone, Logan, UT, USA) supplemented with 15% non-heat-inactivated fetal bovine serum (FBS; Gibco, Grand Island, NY, USA) and 50 U/mL penicillin-streptomycin (Gibco, Grand Island, NY, USA). RS was induced by treating cells with 2 mM HU (Sigma-Aldrich, St. Louis, MO, USA) for 1, 4, or 24 h in complete medium. Recovery was performed by washing treated cells twice with supplemented fresh medium followed by incubation in complete medium for the indicated recovery time. All cells were maintained at 37 °C and 5% CO_2_ and collected at 70–80% confluence.

### 4.2. DNA Damage Induction with Illudin S

Following subculture at ~50% confluence, cells were exposed to increasing concentrations (0, 0.67, 1.67, 2.67, and 4 ng/mL) of Illudin S (Santa Cruz Biotechnology, Dallas, TX, USA) for 72 h in complete DMEM/F12 medium. Cell viability was assessed by MTS assay.

### 4.3. Cell Viability Assay

Cell viability was assessed using the kit CellTiter 96^®^ A_Queous_ One Solution Cell Proliferation Assay (Promega, Madison, WI, USA). Cells were cultured in 96-well plates. After the indicated treatment conditions, the medium was removed and 120 µL of a mix containing supplemented medium (100 µL) and 20 µL of CellTiter 96^®^ A_Queous_ One Solution Reagent ([3-(4,5-dimethylthiazol-2-yl)-5-(3-carboxymethoxyphenyl)-2-(4-sulfophenyl)-2H-tetrazolium; MTS]) and phenazine ethosulfate; PES) was added to each well, gently mixed and incubated at 37 °C for 3 h. Absorbance was measured at 490 nm using a Cytation 3 (BioTek Instruments, Winooski, VT, USA) plate reader.

### 4.4. Indirect Immunofluorescence (IF)

Conventional immunofluorescence (IF) was performed by growing cells on coverslips (Marienfeld Superior™, Lauda-Königshofen, Germany), fixing in 3.7% formalin (Sigma, St. Louis, MO, USA) in PBS for 10 min at room temperature (RT), and permeabilizing with 0.5% Triton X-100 in PBS for 10 min. Blocking was performed in 3% BSA/0.05% Triton X-100 in PBS for 1 h at RT. Primary antibodies were incubated overnight at 4 °C, followed by secondary antibodies for 1 h at RT. DNA was stained with Hoechst 1 µg/mL (Invitrogen, Carlsbad, CA, USA) for 5 min at RT, and slides were mounted with ProLong™ (Invitrogen, Carlsbad, CA, USA). Images were acquired using an OLYMPUS BX53 fluorescence microscope (Olympus Corporation, Tokyo, Japan) and captured using Q-Capture Pro 7 software (QImaging, Surrey, BC, Canada). Quantification of the foci number and nuclear fluorescence intensity was performed from raw, non-processed images using ImageJ software (v1.54; National Institutes of Health, Bethesda, MD, USA) with consistent thresholding and analysis parameters across all conditions. The detail of primary and secondary antibodies used in this work can be found in Supplementary Table S1.

### 4.5. 5-Bromo-2′-deoryuridine Assay

After completion of HU treatment (during the time indicated in the figures), control and HU-treated cells were washed and subsequently incubated with fresh medium containing 20 µM 5-bromo-2′-deoxyuridine (BrdU; Invitrogen, Carlsbad, CA, USA) for 3 h. Next, cells were fixed with 3.7% formalin (Sigma-Aldrich, St. Louis, MO, USA) in PBS. DNA hydrolysis was performed with 2 M HCl (PBS 1×, 0.1% Tween-20) for 15 min at 37 °C, followed by neutralization with a 0.1 M borate buffer (pH 8.5). IF was then performed following the protocol described above, but using 3% BSA/0.01% Tween-20 in PBS as a blocking buffer.

### 4.6. Cell Viability and Apoptosis

Cell viability and apoptosis following HU treatment were assessed simultaneously using the CellTiter-Blue^®^ Cell Viability Assay and the Apo-ONE^®^ Caspase-3/7 Assay kits (Promega, Madison, WI, USA), according to the manufacturer’s instructions, using 96-well plates. Briefly, 20 µL of CellTiter-Blue^®^ Reagent was added to each well to reach a final volume of 120 µL. Plates were gently mixed by manual orbital rotation for 30 s and incubated for 2 h at 37 °C in a cell culture incubator prior to fluorescence measurement (560 nm excitation/590 nm emission). Subsequently, caspase-3/7 activity was measured in the same wells by adding 120 µL of Apo-ONE^®^ Caspase-3/7 Assay Reagent. Plates were gently mixed for 30 s and incubated for 2 h at 37 °C in a cell culture incubator prior to fluorescence measurement (485 nm excitation/527 nm emission). Fluorescence was measured using a Cytation™ 3 plate reader (BioTek Instruments, Winooski, VT, USA).

### 4.7. Propidium Iodide-Based Flow Cytometric Analysis of Cell-Cycle Phase and Sub G1 Population

Cells were harvested, washed with PBS, and fixed by the gradual addition of cold 70% ethanol at −20 °C. Fixed cells were washed in cold PBS, treated with RNase A (10 µg/mL), and stained with propidium iodide (50 µg/mL; Merck, Darmstadt, Germany) in a citrate/NP-40 staining solution to label total DNA content. Samples were kept protected from light and analyzed by FACS-Canto™ A Flow Cytometer (BD Biosciences, San Jose, CA, USA).

### 4.8. Senescence-Associated β-Galactosidase (SA-β-Gal) Staining

SA-β-Gal activity was assessed by cytochemical staining at pH 6.0 as a marker of senescence [56]. Cells were fixed with 3.7% formalin (Sigma-Aldrich, St. Louis, MO, USA) for 5 min at room temperature, washed with PBS, and incubated overnight at 37 °C (without CO_2_) in a staining solution containing X-gal (1 mg/mL; US Biological, Salem, MA, USA), citrate-phosphate buffer (pH 6.0), potassium ferri/ferrocyanide, and magnesium chloride. After staining, cells were washed with PBS and visualized by bright-field microscopy (Leica DM 2500, Leica Microsystems, Wetzlar, Germany). SA-β-Gal-positive cells were identified by the presence of blue staining and quantified by screening the entire well. Positive and negative controls were included to validate the specificity of the assay.

### 4.9. Proximity Ligation Assay (PLA)

Cells grown on coverslips were fixed and permeabilized, then blocked and incubated overnight with primary antibodies against PCNA (mouse) and RNA polymerase II (rabbit). Duolink™ PLUS and MINUS PLA probes (anti-mouse and anti-rabbit) were applied, followed by ligation and rolling-circle amplification using the Duolink™ In Situ Red kit (Merck, Darmstadt, Germany). Fluorescent PLA signals (red dot) were visualized by confocal fluorescence microscopy using a C2+Spectral Confocal Microscope (Nikon Instruments Inc., Tokyo, Japan), and nuclei were counterstained with DAPI. Images were acquired as Z-stacks of 5 optical sections at 0.5 μm step size.

### 4.10. RNA-Sequencing

Total RNA was isolated from ERCC6_WT and ERCC6_K337X cells treated with 2 mM HU for 4 h. This time point was selected to capture acute replication stress-induced transcriptional responses under conditions compatible with cell survival, avoiding extensive apoptosis that would preclude recovery- and selection-based analyses. Untreated controls and time-zero (T0) samples were processed in parallel. A total of ≥100 ng RNA per sample was sent to Novogene Co., Ltd. (Beijing, China) for mRNA library preparation (poly-A enrichment) and sequencing on NovaSeq X Plus (Illumina, San Diego, CA, USA), (PE150; ~12 Gb raw data per sample). An average of >92% clean reads and >11.19 Gb clean bases per sample were obtained, with Q30 values exceeding 96.2%, indicating high sequencing quality. Read alignment was performed using Hisat2 v2.0.5 and gene quantification using featureCounts v1.5.0-p3. Differential expression was analyzed using edgeR (v3.22.5), and genes were considered differentially expressed at a *p* value ≤ 0.05 and |log_2_ fold change| ≥ 1. Reactome pathway enrichment analysis was performed using clusterProfiler v3.8.1, and pathways with a Benjamini–Hochberg–adjusted *p* value ≤ 0.05 were considered statistically significant.

### 4.11. Whole-Exome Sequencing (WES)

Genomic DNA was isolated from ERCC6_WT and ERCC6_K337X cells treated with 2 mM HU for 4 h, as well as from cells treated with 2 mM HU for 4 h followed by 72 h of recovery in fresh medium. Untreated controls and time-zero (T0) samples were processed in parallel. Exactly 1 µg per sample was sent to Novogene Co., Ltd. (Beijing, China) for exome library preparation (Agilent V6; Agilent Technologies, Santa Clara, CA, USA) and sequencing on NovaSeq X Plus (Illumina, San Diego, CA, USA) (PE150; ~12 Gb raw data per sample). WES yielded high-quality data with >93.5% clean reads and >94.2% effective reads, and Q30 values exceeding 95.19%. Target enrichment efficiency resulted in an on-target rate of >68.4%, with an average on-target depth of >134.6×. Exome coverage exceeded 99.4% of target regions, with approximately 95% of bases covered at ≥20×, ~80% at ≥50×, and over 50% at ≥100×, enabling high-confidence variant detection, including low-frequency variants. This sequencing depth supports above 5–10% allele frequency, depending on local coverage. Reads were aligned using BWA to GRCh38, and BAM processing and duplicate marking were performed with Picard. Variant calling for SNVs and Indels was performed using GATK Mutect2 v4.6.2.0 under sensitive detection settings (recover-all-dangling-branches true, min-pruning 0, disable-read-filter MateOnSameContigOrNoMappedMateReadFilter, interval-padding 100, and independent-mates true options). VCF files were normalized and filtered using bcftools v1.21, retaining variants supported by a minimum of five reads. Variant annotation was performed with ANNOVAR v2017 Jun01 using the refGene database.

### 4.12. Statistical Analysis

Statistical tests are indicated in figure legends. Unless otherwise noted, experiments were performed in biological triplicate. Transcriptomic and whole-exome sequencing analyses were conducted using one biological sample per condition, as detailed in the figure legends. Distribution normality and homoscedasticity were assessed prior to parametric testing. A *p* value < 0.05 was considered significant. Analyses were performed using GraphPad Prism 8.0 and RStudio v4.3.2.

Detail of reagents used in this work is provided in Table S1.

### 4.13. Declaration of Generative AI and AI-Assisted Technologies in the Writing Process

While preparing this work the authors used Grammarly (accessed on 7 January 2026), Gemini 2.5 (accessed on 7 January 2026) and ChatGPT 5 (Open AI) to improve the readability and language of the manuscript. After using this tool/service, the authors reviewed and edited the content as needed and take full responsibility for the content of the published article.

## 5. Conclusions

Our findings suggest that TC-NER is essential for TRCs resolution, transcriptional continuity and long-term survival, but may be associated with the preferential retention of protein-coding mutations under mild RS conditions. Understanding this duality is particularly relevant in cancer, where replication stress and transcriptional hyperactivation coexist and may influence the genomic distribution of somatic mutations.

Although immortalized primary fibroblasts provide a controlled human model for studying initial mutagenesis events, responses in tumor-derived cells may differ. We hope that our findings encourage further studies exploring how ERCC6 shapes cellular responses to replication stress in additional systems and contexts.

## Data Availability

The original contributions presented in this study are included in the article/Supplementary Materials. Further inquiries can be directed to the corresponding author.

## Supplementary Materials

The following supporting information can be downloaded at: https://www.mdpi.com/article/10.3390/ijms27031154/s1.

## Abbreviations

ERCC6	Excision Repair Cross-Complementation Group 6
CSB	Cockayne Syndrome Group B protein
CS	Cockayne Syndrome
RS	Replication Stress
TC-NER	Transcription-Coupled Nucleotide Excision Repair
TRCs	Transcription–Replication Conflicts
DSBs	DNA Double-Strand Breaks
HR	Homologous Recombination
NHEJ	Non-Homologous End Joining
Alt-EJ	Alternative End Joining
BIR	Break-Induced Replication
GG-NER	Global Genome Nucleotide Excision Repair
ATM	Ataxia Telangiectasia Mutated kinase
CDK2	Cyclin-Dependent Kinase 2
PARP1	Poly(ADP-ribose) Polymerase 1
PARP2	Poly(ADP-ribose) Polymerase 2
RPA	Replication Protein A
pRPA	Phosphorylated Replication Protein A
γH2AX	Phosphorylated Histone H2AX (Ser139)
53BP1	p53-Binding Protein 1
RNAPII	RNA Polymerase II
PCNA	Proliferating Cell Nuclear Antigen
PLA	Proximity Ligation Assay
HU	Hydroxyurea
BrdU	5-bromo-2′-deoxyuridin
