# Supplementary material for: Transcription-Coupled Repair Promotes the Retention of Mutations in Coding Regions During Replication Stress
Source: Int J Mol Sci. 2026 Jan 23;27(3):1154. doi: 10.3390/ijms27031154 (PMC12896993; doi:10.3390/ijms27031154)
Supplement: Supplementary file 1 [file ijms-27-01154-s001.zip › 1. Zambrano et al., Supplemental_Information.pdf]

Supplemental Information

Supplementary Figure S1. Illudin S is selectively lethal in ERCC6-deficient cells. Cell viability of ERCC6_WT (blue circles) and ERCC6_K337X (pink squares) fibroblasts treated with increasing concentrations of Illudin S, measured by MTS assay. Viability is expressed as absorbance normalized to untreated controls. Data represent mean ± SD of three independent experiments (n=3). Statistical analysis: two-way ANOVA followed by Sidak's post hoc test; $p < 0.0001$ (****).

Supplementary Figure S2. Comparable basal RPA signal in ERCC6_WT and ERCC6_K337X cells. **(A)** Nuclei are shown in grey and RPA in red. Scale bar, 50 μm. **(B)** Integrated nuclear signal density (IntDen) for RPA. Box-and-whisker plots follow the Tukey method. Data represent mean ± SD from three independent experiments (n > 180 nuclei). Statistics: Welch's ANOVA with Brown–Forsythe correction, followed by Games–Howell post hoc test. Significance level: $p < 0.0001$ (****).

Supplementary Figure S3. Lack of ERCC6 impairs the resolution of TRCs under HU, showing an increase in PLA-detected conflict signals. **(a)** Proximity Ligation Assay (PLA) detecting RNAPII–PCNA interactions in ERCC6_WT and ERCC6_K337X cells under control and HU (4 h). Nuclei are shown in blue (DAPI) and PLA foci in red. Scale bar, 50 μm. **(b)** Quantification of PLA foci per nucleus. **(c)** Median PLA focus volume per nucleus for the same conditions. Data represent mean ± SD from three independent experiments, 4-5 fields each (n=12-15 images). Statistical analysis: two-way ANOVA followed by Sidak's post hoc test; ns = not significant, $p < 0.01$ (**) and $p < 0.001$ (***).

Supplementary Figure S4. Comparative differential gene expression across ERCC6 genotypes and replication stress conditions. Bar plots summarizing total DEGs (all), upregulated (up) and downregulated (down) genes across the indicated pairwise comparisons. Gene expression levels were quantified using FPKM values. DEGs were identified using edge R (p value < 0.05).

Supplementary Figure S5. Basal transcriptional differences associated with ERCC6 status. **(a)** Volcano plot showing differential gene expression between ERCC6_WT and ERCC6_K337X cells under basal (untreated) conditions. Log_2 fold change is plotted against $-log_{10}$(p value); significantly regulated genes (p value < 0.05) are in red. Genes highlighted are differentially expressed genes upon HU treatment in both ERCC6_WT and ERCC6_K337X cells. Differential expression was determined using edgeR with

pseudoreplicates (n=1 per condition). **(b)** Reactome pathway enrichment analysis of significantly DEGs under basal conditions (ERCC6_WT_Control vs ERCC6_K337X_Control). Reactome significance threshold: adjusted *p* adj (BH correction) <0.05. Pathway-level analyses were performed using pseudoreplicates (n = 1 per condition).

Supplementary Figure S6. Distribution of SNVs and INDELs called in RNA, by impact and genomic region after HU exposure. **(a–b)** Δ number of mutations (HU 4 h – Ctrl 4 h) for SNVs, **a)** and INDELs, **b)**, classified by predicted functional impact (HIGH, MODERATE, LOW, MODIFIER). **(c–d)** Δ mutation counts across genomic regions (DOWN/UP-STREAM, EXON, INTRON, SPLICE SITES, UTRs). Variants were called from RNA-seq data.

Supplementary Table S1. Reagents and antibodies used in this study.
