# Supplementary material for: Transcription-Coupled Repair Promotes the Retention of Mutations in Coding Regions During Replication Stress
Source: Int J Mol Sci. 2026 Jan 23;27(3):1154. doi: 10.3390/ijms27031154 (PMC12896993; doi:10.3390/ijms27031154)
Supplement: Supplementary file 1 [file ijms-27-01154-s001.zip › 8. Supplementary_Table_S1.pdf]

Primary antibody name	Manufacturer	ID	Host	Target Species	Dilution
Anti-BrdU antibody [BU1/75 (ICR1)]	Abcam	ab6326	Rat	Human	1:250
Anti-RPA32/RPA2 antibody [9H8]	Abcam	ab2175	Mouse	Human	1:250
Phospho RPA32 (S33)	Bethyl	A300-246A	Rabbit	Human	1:500
Anti-phospho-Histone H2A.X (Ser139), clone JBW301	Merck	# 05-636	Mouse	Human	1:250
53BP1	Cell Signaling	#4937	Rabbit	Human	1:250
PCNA Monoclonal Antibody (PC10 (3F81))	Invitrogen	#14-9910-82	Mouse	Human	1:200
Anti-RNA polymerase II CTD repeat YSPTSPS (phospho S2) antibody [EPR18855]	Abcam	ab193468	Rabbit	Human	1:150
**Secondary antibody name**					
Alexa Fluor 594	Thermo Fisher Scientific	A-11005	Goat	Mouse	1:2000
Alexa Fluor 488	Thermo Fisher Scientific		Goat	Mouse	1:2000
Alexa Fluor 594	Thermo Fisher Scientific	A-11012	Goat	Rabbit	1:2000
Alexa Fluor 488	Thermo Fisher Scientific	A-11008	Goat	Rabbit	1:2000
Alexa Fluor 594	Thermo Fisher Scientific		Goat	Rat	1:250
**Others**					
5-bromo-2'-deoryuridine	Invitrogen	B23151			
Apo-ONE Homogeneous Caspase-3/7 Assay	PROMEGA	G7791			
CellTiter-Blue Cell Viability Assay	PROMEGA	G8081			
E.Z.N.A Total RNA Kit I	omega BIO-TEK	R6834-02CH			
Hydroxyurea	Sigma-Aldrich	H8627-1G			
Illudin S	Santa Cruz	SC-391575			
QUICK-DNA^{TM} MiniPrep	ZYMO RESEARCH	D3024			
ProLongTM Glass Antiface Mountant	Invitrogen	P36980			
CellTiter 96® AQueous One Solution Cell Proliferation Assay (MTS)	PROMEGA	G3581			
Duolink PLA Starter Kit Red	Merck	DUO92101-1KT			
X-Gal	US BIOLOGICALS	UB.X1000-1G			
Propidium iodide	Merck	81845-25MG			
