# Supplementary figures and images for: Transcription-Coupled Repair Promotes the Retention of Mutations in Coding Regions During Replication Stress
Source: Int J Mol Sci. 2026 Jan 23;27(3):1154. doi: 10.3390/ijms27031154 (PMC12896993; doi:10.3390/ijms27031154)

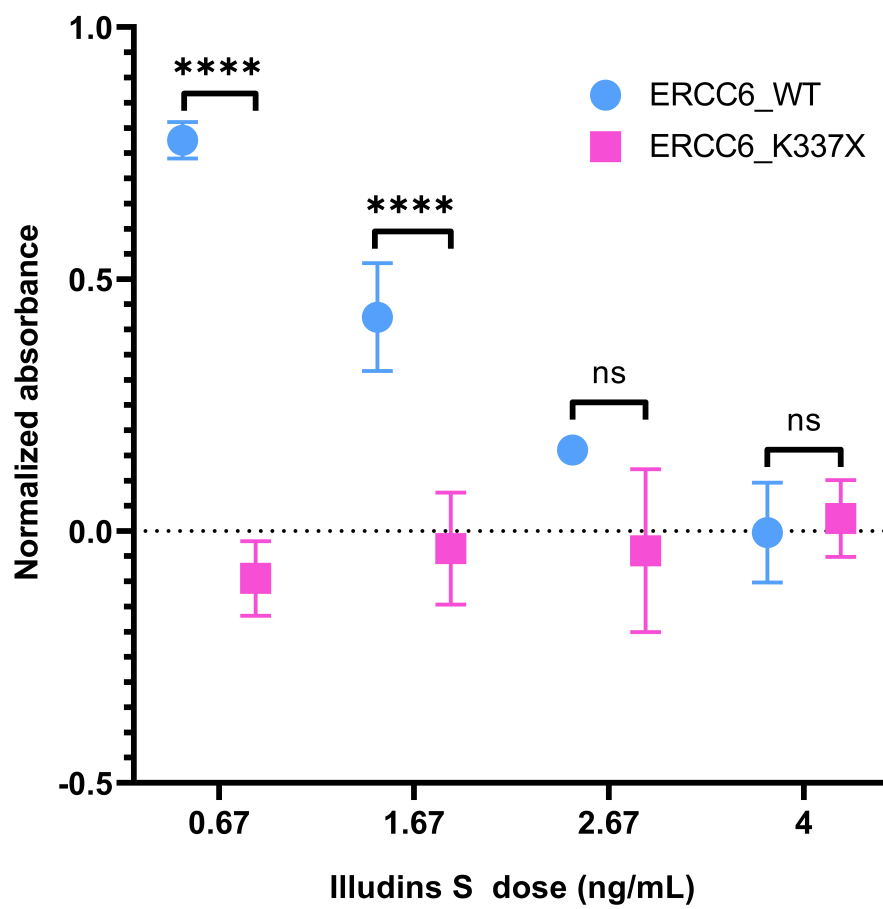

Supplement: Supplementary file 1 [file ijms-27-01154-s001.zip › 2. Supplementary_Figure_S1.pdf]

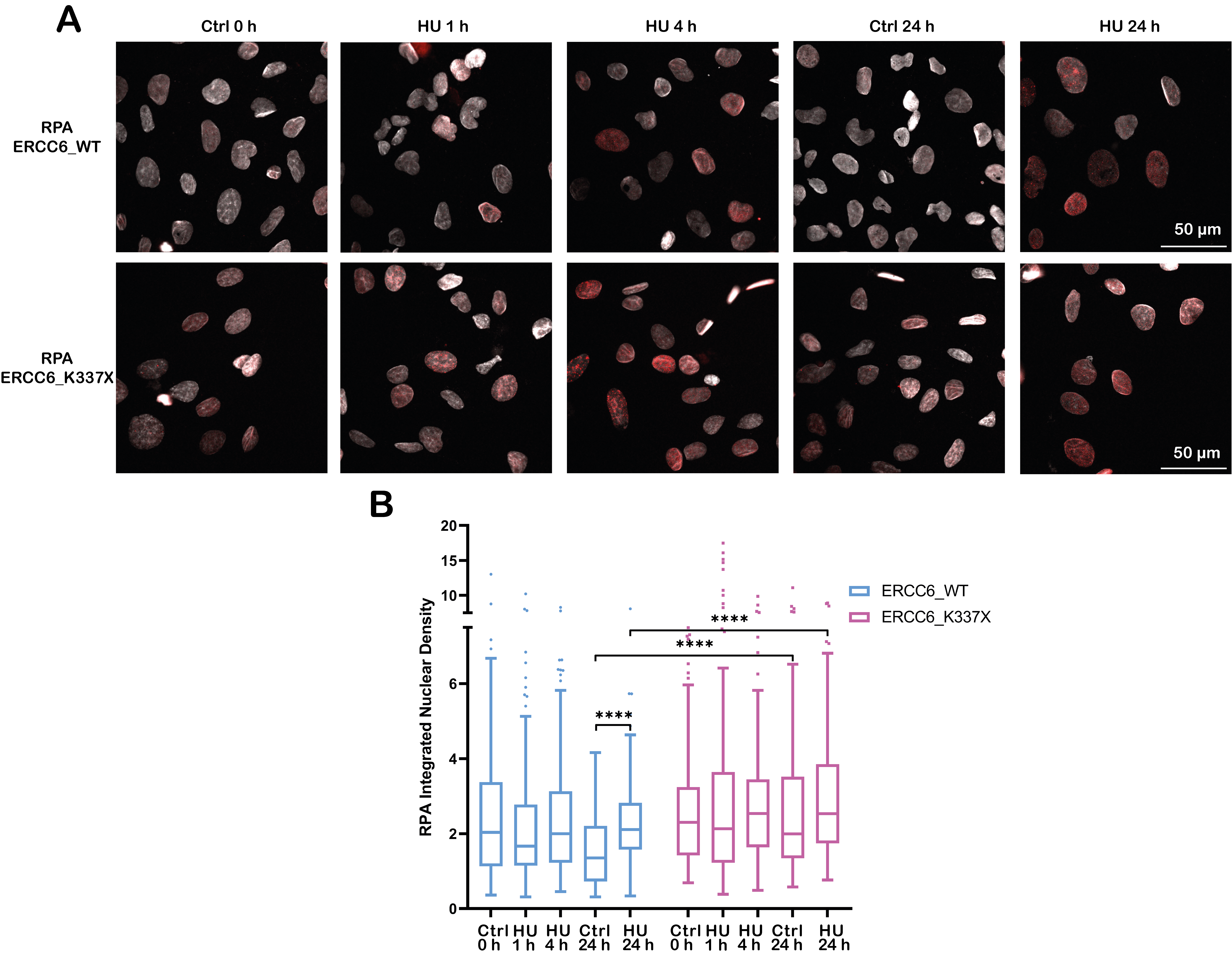

Supplement: Supplementary file 1 [file ijms-27-01154-s001.zip › 3. Supplementary_Figure_S2.png]

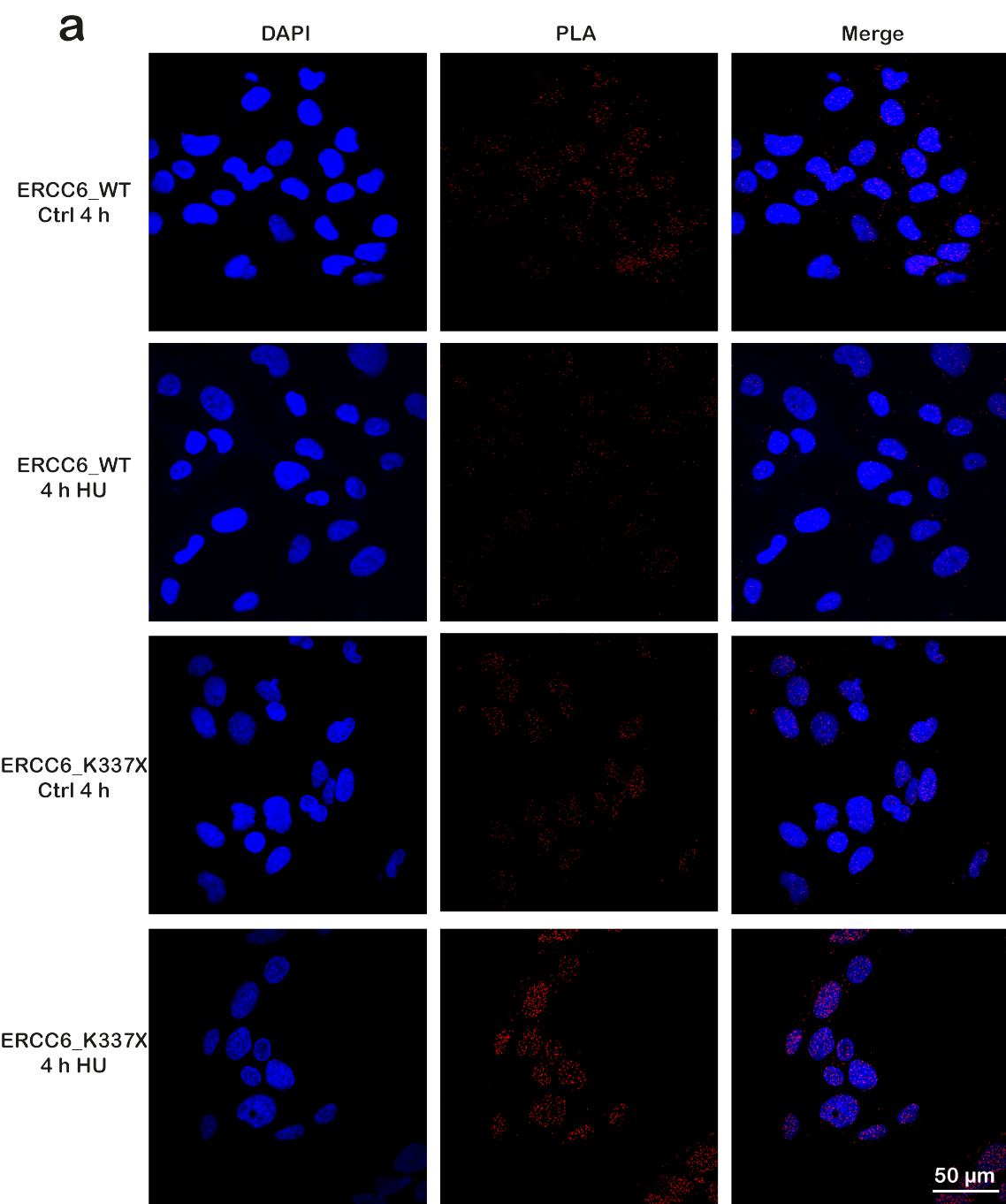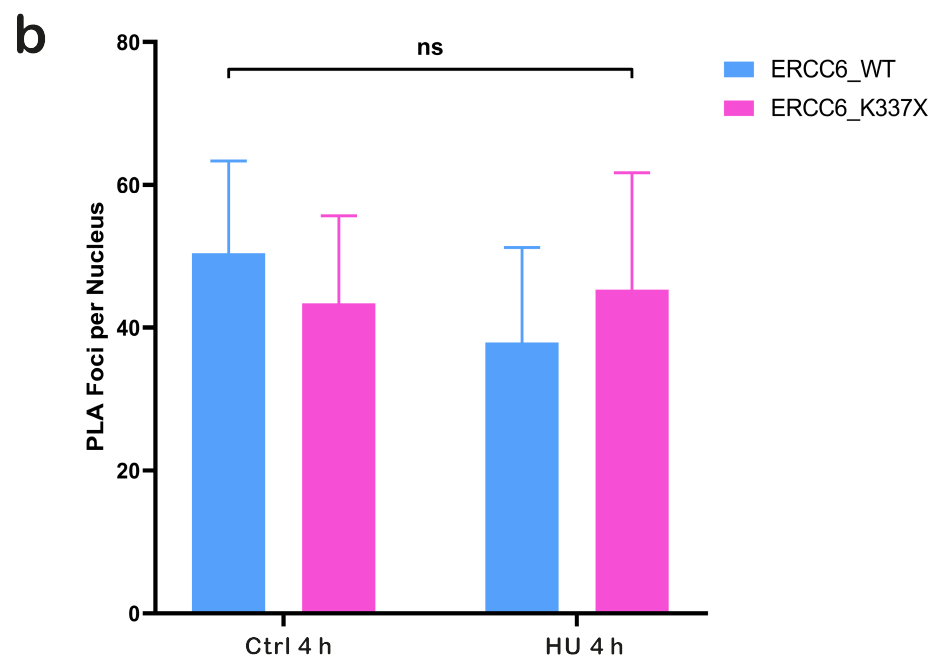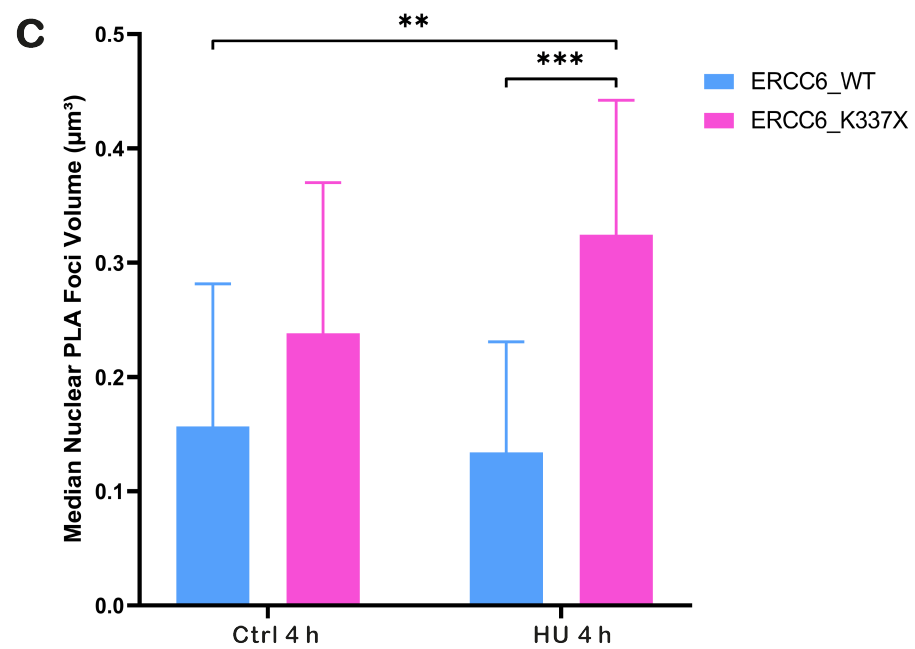

Supplement: Supplementary file 1 [file ijms-27-01154-s001.zip › 4. Supplementary_Figure_S3.pdf]

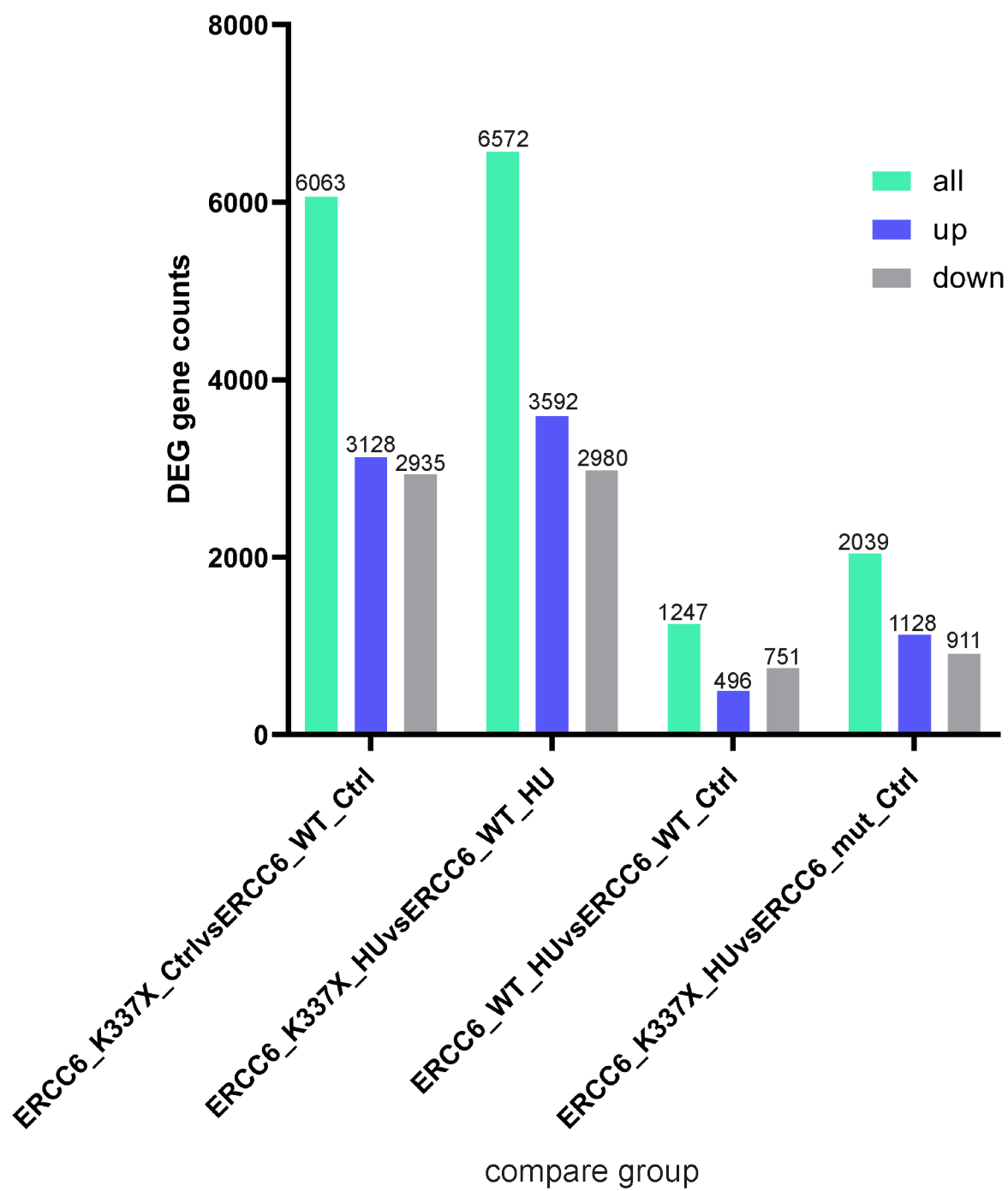

Supplement: Supplementary file 1 [file ijms-27-01154-s001.zip › 5. Supplementary_Figure_S4.pdf]

a

ERCC6\_WT\_Control vs ERCC6\_K337X\_Control

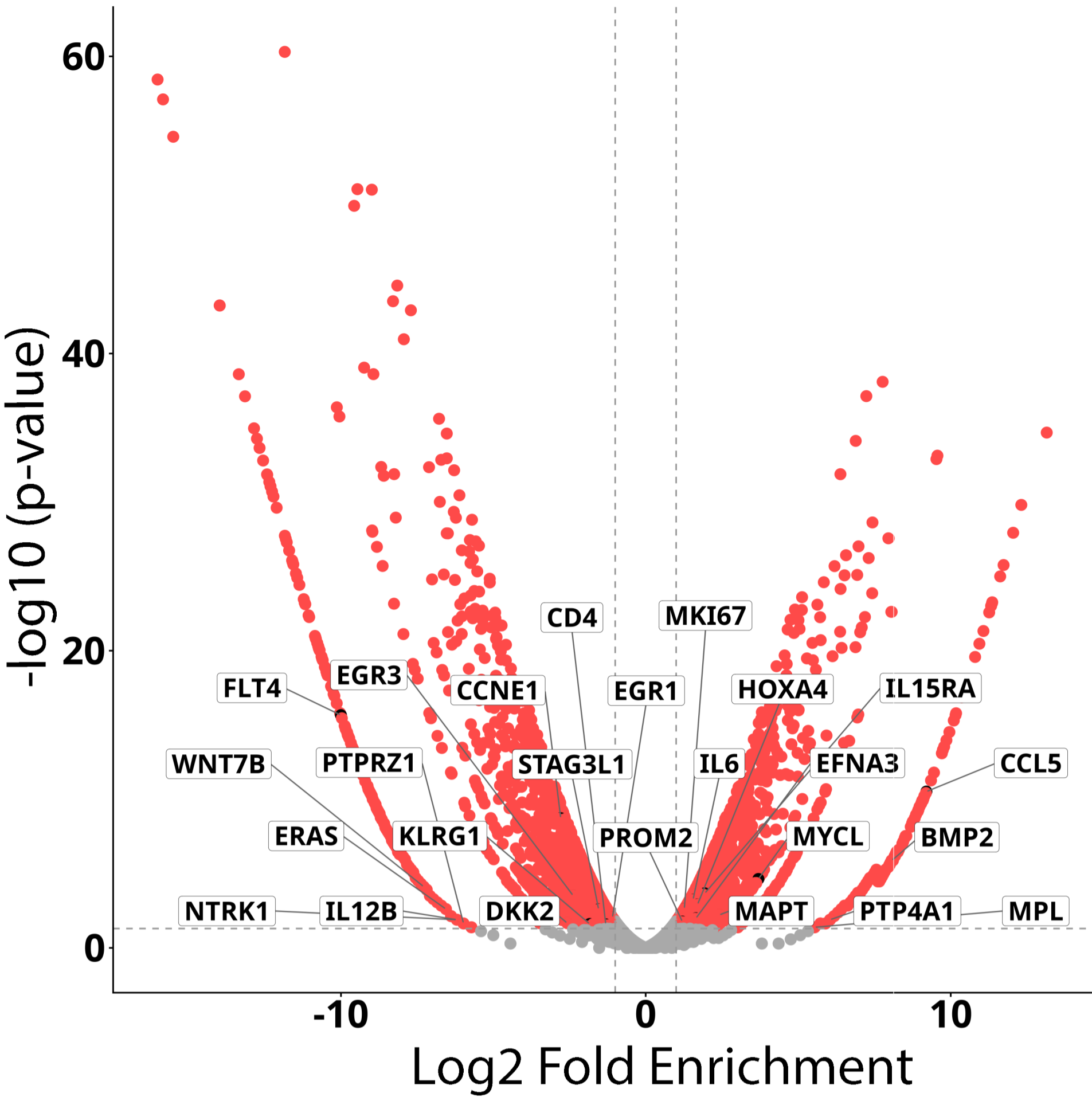

b

ERCC6\_WT\_Control vs ERCC6\_K337X\_Control

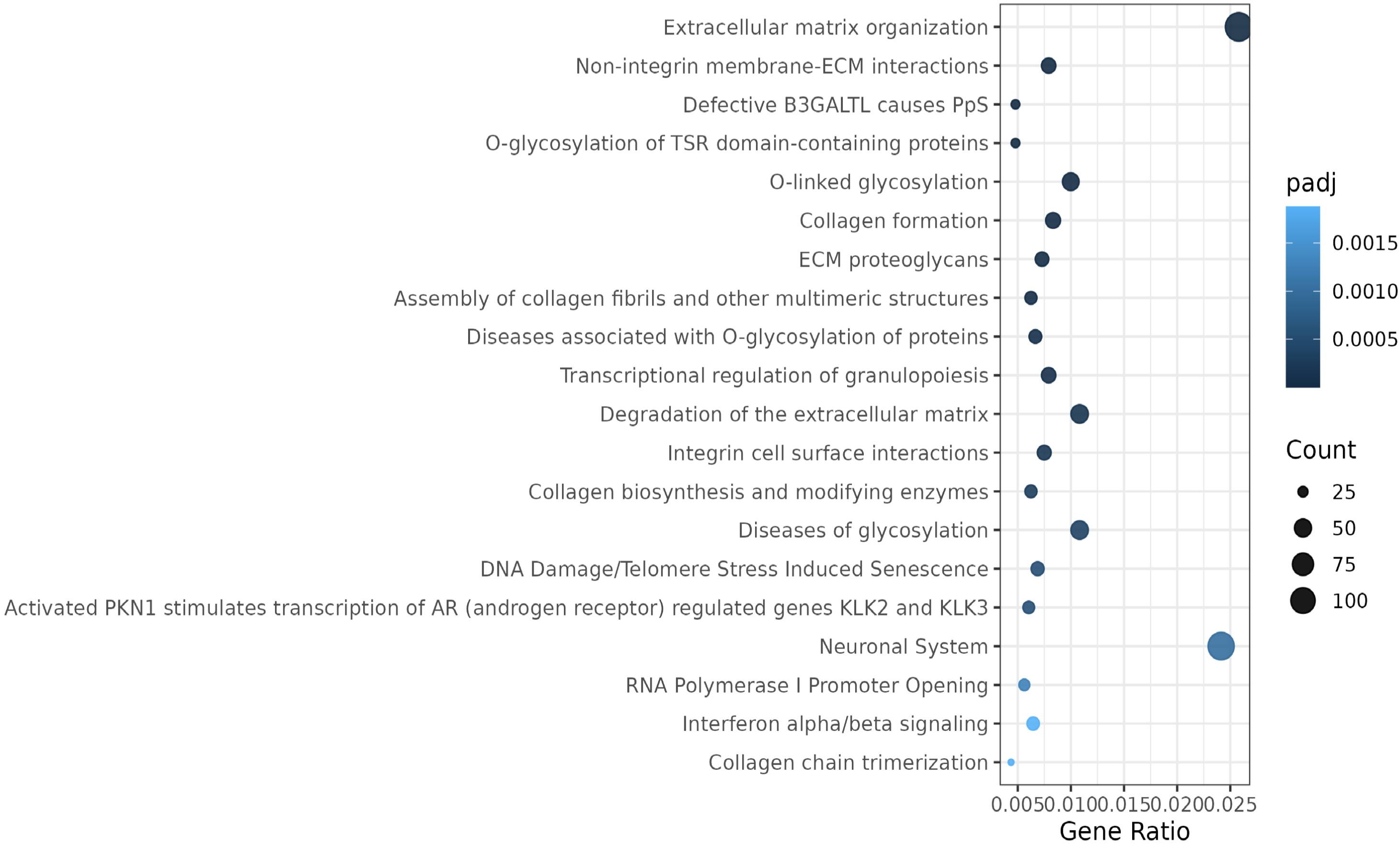

Supplement: Supplementary file 1 [file ijms-27-01154-s001.zip › 6. Supplementary_Figure_S5.pdf]

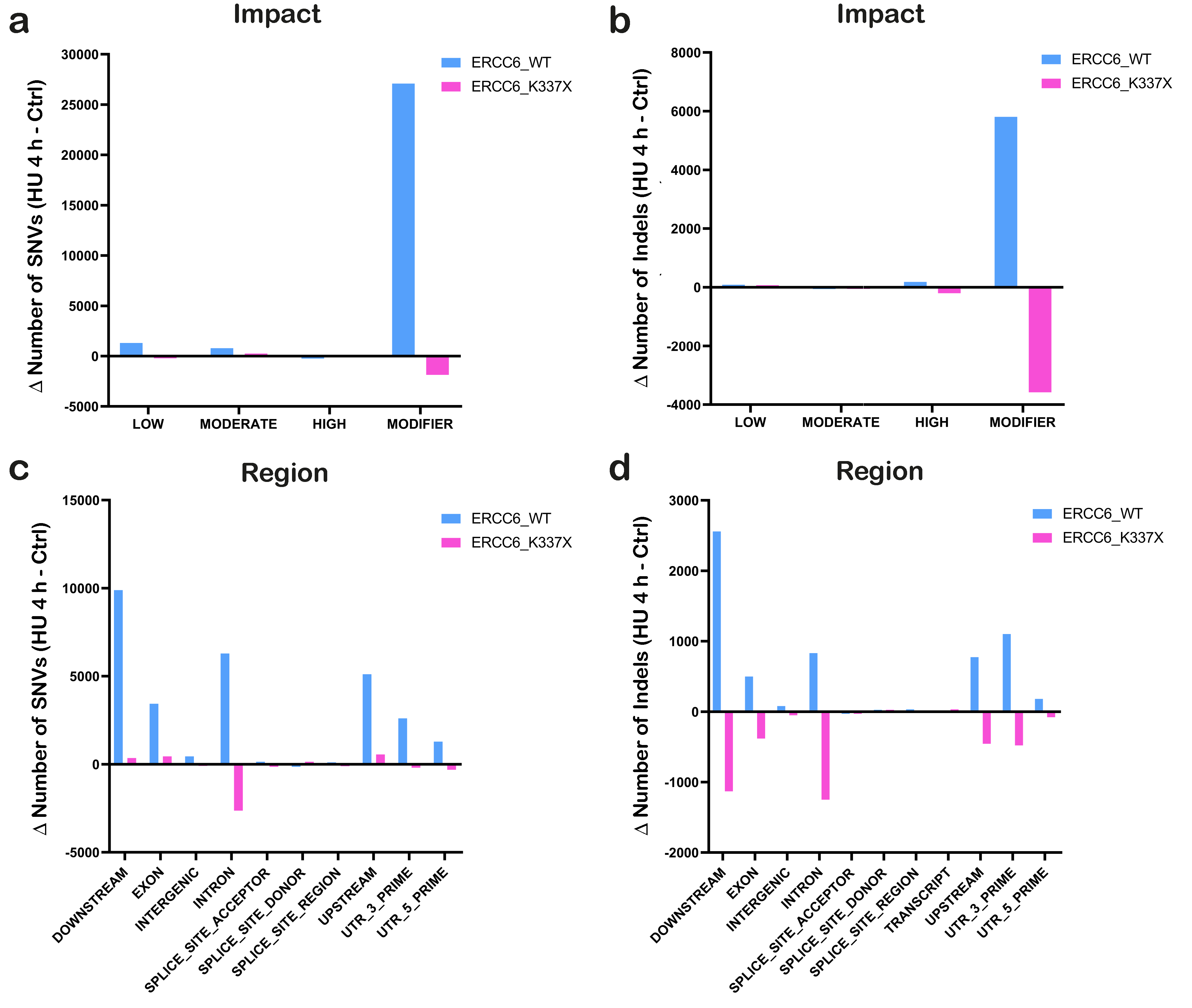

Supplement: Supplementary file 1 [file ijms-27-01154-s001.zip › 7. Supplementary_Figure_S6.png]
